# Human *Metapneumovirus* as a Cause of Community-Acquired Respiratory Illness^1^

**DOI:** 10.3201/eid0809.020084

**Published:** 2002-09

**Authors:** Joanne Stockton, Iain Stephenson, Douglas Fleming, Maria Zambon

**Affiliations:** * Public Health Laboratory Service Central Public Health Laboratory, London, United Kingdom; † Leicester Royal Infirmary, Leicester, United Kingdom; ‡ Royal College of General Practitioners Surveillance Unit, Birmingham, United Kingdom

**Keywords:** Human *Metapneumovirus*, community, acute respiratory illness

## Abstract

Human *metapneumovirus* (HMPV) is a recently identified *Paramyxovirus* first isolated from hospitalized children with acute respiratory tract infections (ARTI). We sought evidence of HMPV infection in patients who had visited general practitioners, had influenzalike illnesses (ILI), and had negative tests for influenza and *Human respiratory syncytial virus* (HRSV). As part of national virologic surveillance, sentinel general practices in England and Wales collected samples from patients of all ages with ILI during winter 2000–01. Reverse transcriptase-polymerase chain reaction (PCR) for HMPV, influenza A (H1 and H3), influenza B, and HRSV was used to screen combined nose and throat swabs. PCR products from the HMPV-positive samples were sequenced to confirm identity and construct phylogenetic trees. Of 711 swabs submitted, 408 (57.3%) were negative for influenza and HRSV; HMPV was identified in 9 (2.2%) patients. HMPV appears to be associated with community-acquired ARTI. The extent of illness and possible complications related to this new human virus need to be clarified

Despite control of many infectious diseases in the industrialized world, acute viral respiratory tract infections (ARTI) remain a leading cause of illness. Although usually self-limiting in healthy adults, these infections are responsible for a substantial loss of productive time and are important factors in the illness and death of the elderly population. Various genetically diverse viruses, often with multiple types, may cause respiratory illness; of these, influenza receives the greatest attention (1). *Human respiratory syncytial virus* (HRSV) is also increasingly implicated as an important pathogen (2).

The association between the incidence of ARTI and excess winter deaths in the United Kingdom is well recognized (1). Regression modeling associates excess winter deaths with influenza and HRSV but also suggests that other pathogens may be involved (3).

Studies of the impact of respiratory virus infections are limited by difficulty in distinguishing respiratory pathogens clinically and in the laboratory (4,5). Despite improved sensitivity with diagnostic techniques such as reverse transcriptase-polymerase chain reaction (RT-PCR), approximately 40% of specimens from patients with community-acquired respiratory illnesses during peak winter months contain no identified viral pathogen (2,5,6).

A new pneumovirus, Human *Metapneumovirus* (HMPV), has recently been isolated in the Netherlands (7). The *Pneumovirinae* subfamily is classified into *Pneumovirus*, containing HRSV, and *Metapneumovirus* genera. In 2001, Van den Hoogen et al. (7) reported the detection of HMPV in nasopharyngeal aspirates taken in a 10-year period from 28 hospitalized children and infants with respiratory tract infections who had signs and symptoms similar to those of HRSV infection.

Establishing sensitive methods for virus detection helps to clarify the relative contribution of different pathogens to the extent of illness in the community. This information is important for future development of specific antiviral therapies and vaccines. We examined specimens submitted from patients seen in general practice with influenzalike illnesses (ILI) during winter 2000–01 to detect HMPV as a possible cause of influenza- and HRSV-negative ILI.

## Materials and Methods

### Sentinel General Practice Networks

Clinical episodes of ILI are recorded by continuous monitoring in approximately 75 sentinel practices in England and Wales, covering a population of 700,000. New episodes of illness are noted and weekly returns submitted to the Royal College of General Practitioners research unit. ILI was defined as symptoms of fever, cough, and muscle pains with duration of <5 days (8,2). Virologic surveillance of ILI is performed by a subset of the sentinel practices. Combined nose and throat swabs are taken from persons diagnosed with ILI at the time they see a clinician, which is often several days after onset of illness. Swabs are mailed in virus transport medium to the Central Public Health Laboratory for analysis. Samples are divided into aliquots, labeled, and then frozen at –80°C on receipt.

### HRSV and Influenza Detection

Detection of HRSV and influenza was performed prospectively on receipt of samples by using multiplex PCR as previously described (2,8).

### HMPV Detection

Stored nucleic acid from 348 samples negative for influenza and HRSV was analyzed by using a PCR for HMPV (7). No optimization of the PCR detection was undertaken. Detection of HPMV was based on primers located in the L gene. For a further 60 (14.8%) samples from which the nucleic acid stored was insufficient, an aliquot of the original clinical material was re-extracted by using a Magnapure (Roche Diagnostics GmbH, Mannheim, Germany) automated extraction machine according to the manufacturer’s instruction; RT was performed as described previously (8). Twenty microliters of cDNA was added to 80 μL of reaction mix, which yielded a final concentration of 20 mM Tris-HCl pH8.4, 10 mM MgCl_2_, 50 mM KCl, and 3 U *Taq* polymerase and 50 pmol of each primer. Amplification, using a DNA Engine thermocycler (MJ Research, Essex, England), consisted of 1 cycle at 94°C for 2 min, followed by 35 cycles of 94°C 1 min, 58°C 1 min, and 72°C for 1 min. Amplicons of the expected size (171 bp) were visualized by agarose gel electrophoresis. PCR sensitivity was determined by cloning of the PCR product into TOPO PCR4 vector (Invitrogen Corp., San Diego, CA) according to the manufacturer’s instructions. Plasmid preparations were performed by using a Qiagen minprep kit (QIAGEN, Inc., Valencia, CA), according to the manufacturer’s instructions. DNA quantitation followed by PCR, using the original primers and conditions (as above) with the addition of 0.1 mM of each deoxynucleoside triphosphate per reaction, was undertaken using the plasmid plus insert as template. Total RNA at the time of extraction was estimated by spectrophotometry at A_260_.

The resulting amplicons were sequenced by using a Ceq Dye terminator sequencing kit (Beckman Coulter, Inc., Somerset, NJ) according to the manufacturer’s instructions. Sequenced reactions were run on a CEQ 2000 capillary sequencer (Beckman Coulter). Resulting sequence was BLAST searched; 98% identity to deposited sequences of HMPV was seen.

### Phylogenetic Analysis

Sequences were aligned by using the program Megalign (Lasergene, DNA STAR, Inc., Madison, WI) and exported into PAUP*(URL: http://paup.csit.fsu.edu/about.html). Maximum likelihood trees were created by using the K81 model of evolution and bootstrapped x1000.

## Results

### ILI Clinical Diagnosis

A total of 711 swabs were submitted from cases of ILI seen at the subgroup of 16 sentinel practices supplying virologic specimens. These cases constituted approximately 65% of the total consultations of ILI in these practices from October 1, 2000, through March 30, 2001, covering a population of 241,000. Swabs were obtained from all age groups: 79 (11%) from those <5 years; 115 (16%) from those 5–14.9 years; 300 (42%) from those 15–39.9 years; 179 (25%) from those 40–64.9 years; and 37 (5%) from those >65 years; for 1 sample the age was unknown.

### HMPV PCR Sensitivity

The sensitivity of the PCR was determined to be at least 1 femtogram of DNA template and 0.32 μg of a total RNA preparation.

### HMPV

**Figure 1:**
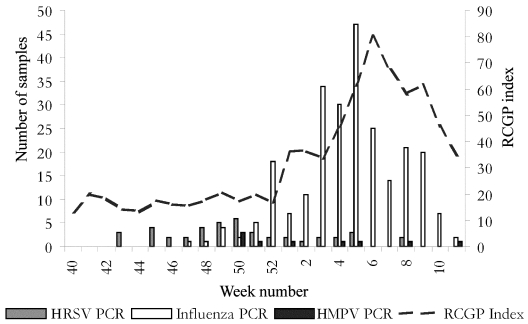
Incidence of influenzalike illness consultations in England during winter 2000–01 and timing of collection of positive samples for human *Metapneumovirus* (HMPV). HRSV, *Human respiratory syncytial virus*; PCR, polymerase chain reaction; RCGP, Royal College of General Practitioners.

A total of 408 specimens were negative for influenza and HRSV; of these, 405 (99.3%) samples were available for analysis and examined for HMPV by RT-PCR. Nine (2.2%) of these were positive for HMPV by PCR. Samples were unrelated geographically or epidemiologically. Clinical history and findings are summarized in the table. HMPV was detected in samples from a child <1 year old, from four persons ages 18–64 years, and four persons ages >65 years. Six (67%) of the nine had clinical evidence of lower respiratory tract involvement. Four (44%) received antibiotic therapy at the time they were seen by a clinician. All made a complete recovery. Figure 1 shows timing of HMPV-positive sample collection relative to the circulation of HRSV and influenza, and the rate of clinical ILI consultations throughout the study period.

**Table:** Clinical information on patients with influenzalike illness and positive *Metapneumovirus* polymerase chain reaction results, seen by general practitioners^a^

Case/sex	Age (yr)	Past medical history	Influenza vaccine	Clinical signs and symptoms (days symptoms persist/total days ill)
F	46	None	No	(7/7) febrile respiratory symptoms, sore throat, malaise, and lethargy. Chest clear
F	20	None	No	(2/7) sore throat, unproductive cough, sternal pain, wheeze. Signs: 37.4°C; chest clear
M	1	None	No	(4/7) coughing, vomiting. OE: 37.2°C, chest and abdomen normal
F	75	Mild hypertension	Yes	(4/7) febrile respiratory illness, cough. Signs: 37.4°C, bilateral basal crackles
F	57	COPD^b^	Yes	(5/7) coryza, sore throat, thick green sputum. Signs: poor air entry and bilateral crackles
M	65	Mild hypertension	No	(3/7) cough, upper respiratory symptoms. Chest clear
F	73	None	Yes	(6/7) cough, green sputum, and dyspnea
M	74	COPD^b^ IHD^b^	Yes	(5/7) cough, malaise, sputum, breathlessness
M	46	None	No	(6/7) days sore throat, sputum, wheeze, breathlessness Signs: PEFR^b^ 260 mL/min, wheeze

^a^All made a full recovery. ^b^COPD, chronic obstructive pulmonary disease; IHD, ischemic heart disease; OE, on examination; PEFR, peak expiratory flow rate.

### HMPV Phylogeny

**Figure 2:**
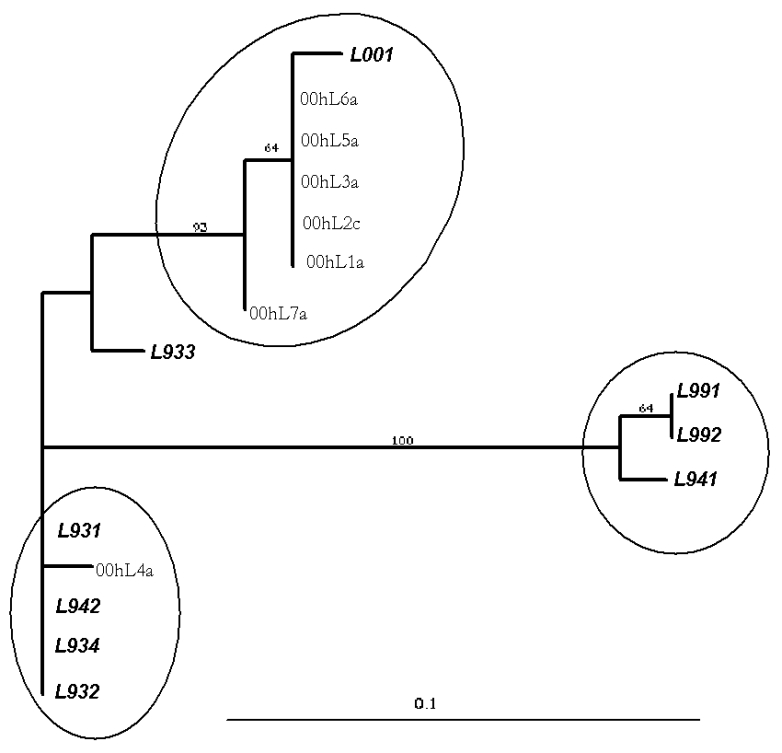
Phylogenetic tree showing sequence analysis of human *Metapneumovirus* (HMPV). Isolates prefixed with L were obtained from GenBank and represent isolates from the Netherlands. Isolates prefixed with 00hL are from this study; the following number indicates strain designations throughout the season: a, sample from an adult; c, sample from a child (<15 years). Scale shown is proportional to number of nucleotide substitutions per site.

The phylogenetic analysis of the L gene sequences from our patients and nine previously reported (7) showed at least two possible clusters of sequences, which may provide evidence for at least two groups of HMPV. The sequences obtained from the adult patients cluster with those obtained from children from our study and those previously reported (7) (Figure 2).

## Discussion

The extent of illness in the community caused by circulating respiratory viruses is currently underestimated. Problems in diagnosing these viruses include capture of suitable specimens for virologic diagnosis, limitations caused by transport of labile viruses, and sampling from adult subjects, who may shed fewer viruses than children (9). However, PCR is more sensitive for detecting a range of respiratory viruses than are culture or serologic methods and has been shown to be a robust diagnostic tool (10).

We have found evidence of HMPV in approximately 2.2% of cases of ILI that were negative for HRSV and influenza. If no other HMPV were detected in the rest of the samples (i.e., as a dual infection with either HRSV or influenza), the overall detection rate in this population would be 1.3% with the use of the PCR method. We cannot exclude the possibility that HMPV is carried asymptomatically in the human respiratory tract and is of no clinical importance, nor the possibility of dual infection. Our estimate, therefore, is likely to be a minimum one. Respiratory virus coinfections appear to occur at a rate of 1% to 3% in various sample sets (2,11). Nonetheless, as the specimens are taken from symptomatic patients seeking medical consultation at the time of respiratory illness, the assumption that the pathogen detected is responsible for the illness is reasonable.

When HMPV was first isolated, it was associated with symptoms of ARTI in infants and children similar to those seen in HRSV infection, which ranged in severity from self-limiting mild respiratory illness to respiratory failure requiring ventilation. Therefore, diagnosing HMPV infection and differentiating it from other respiratory viruses may be impossible on clinical grounds alone. All the specimens were negative for other respiratory viruses, and it was concluded that HMPV was likely to be the responsible pathogen.

Our results are consistent with the association of HMPV with acute respiratory infections in winter (7) and demonstrate that this virus is associated with at a least a proportion of mild, community-acquired, self-limiting respiratory illnesses in all age groups. Initial seroprevalence data documented that all children were seropositive by 5 years of age (7), and our results imply that, like HRSV, HMPV is capable of causing clinically important reinfection in late childhood or adult life.

We consider that our data on the prevalence of HMPV in a sentinel physician surveillance scheme, used for combined clinical-virologic monitoring, are likely to be a minimum estimate of this virus’ contribution to acute respiratory infections, for several reasons. Our detection strategy depends on the use of PCR targets in the L gene of a paramyxovirus and has been determined by sequence availability. A limitation of the current study may be the sensitivity of the PCR used. Although the sensitivity was determined for a DNA target and total RNA preparation, no information is currently available, for comparison, on the sensitivity of the PCRs that other studies have used (12,13). Nethertheless, we consider these estimates may be taken as a robust minimum value. A differential gene transcription present in the *Paramyxoviridae* as a whole suggests that targeting diagnostics to genes that are transcribed more proximally may be a more sensitive approach. As virus quantitiation methods are developed and in vitro transcription methods are established, determination of copy number sensitivity will become possible. Currently, no methods for virus quantitation in cell culture are established, and the full genome sequence has only very recently been published (14). Furthermore, no information exists on the efficiency of HMPV detection directly from nose and throat swab specimens, without culture amplification as was done in the Dutch study (7). As only patients presenting with ILI symptoms were sampled in this surveillance cohort, most acute respiratory infections that occur outside this surveillance definition are unsampled, thereby increasing the likelihood of underestimation of HMPV as a cause of all respiratory infections. Since sampling only occurs during the winter season (October–March), we cannot determine whether the virus circulates year-round or whether it has seasonal peaks during the summer or winter months. Clearly, HMPV cocirculates with influenza and HRSV (Figure 1, data from this national surveillance program published on the PHLS web [URL: http://www.phls.co.uk/topics_az/influenza/Activity0102/graph12.pdf], which adds to the potential for clinical diagnostic confusion.

 Four out of five ILI cases in adults <65 years of age or with cardiorespiratory conditions in which HMPV was detected occurred in persons who had received influenza vaccination in accordance with national vaccination policy. This finding indicates that HMPV infection may cause an illness difficult to distinguish from influenza in the elderly and may be one of the reasons for underestimating the clinical efficacy of influenza vaccine in the elderly when clinical endpoints are used.

Sequencing analysis was performed on seven out of nine of our isolates by using primers that amplify a portion of the L gene. This analysis identified at least two possible strain clusters. By analogy with other paramyxoviruses, the L gene codes for the viral polymerase. This gene typically shows less variability than genes coding for surface proteins. These results are consistent with the limited published evidence for genetic variation between HMPV isolates, suggesting the possibility of more than one type (7). Analysis of the structural membrane proteins that are more commonly associated with genetic or antigenic drift may identify further variability. Accurately determining relationships between strain clusters may also require sequencing of larger portions of the genome.

This study has identified, for the first time, HMPV in adults and children in the community. Phylogenetic analysis confirmed that similar strains are circulating in adults and children at the same time, a feature also seen with influenza and HRSV infection (2).

More sensitive diagnostic tools need to be developed to ascertain a true estimate of the extent of illness caused by HMPV in the general community.

Although ILI and ARTI are common illnesses and usually self-limiting in healthy persons, these infections have a major impact on the overall health of the population with substantial loss of productive time. Continuous surveillance of respiratory pathogens is important for public health. Assessing the role of individual pathogens is important for the potential development of vaccines and for considering specific antiviral therapy. In the future, we will consider including HMPV detection in our routine surveillance programs of respiratory illness. Whether this interesting new virus, HMPV, plays a major clinical role in winter hospital admissions and excess deaths in different age groups of the general population remains to be seen.
